# Viral suppression in adults, adolescents and children receiving antiretroviral therapy in Cameroon: adolescents at high risk of virological failure in the era of “*test and treat*”

**DOI:** 10.1186/s12981-019-0252-0

**Published:** 2019-11-19

**Authors:** Joseph Fokam, Samuel Martin Sosso, Bouba Yagai, Serge Clotaire Billong, Rina Estelle Djubgang Mbadie, Rachel Kamgaing Simo, Serge Valery Edimo, Alex Durand Nka, Aline Tiga Ayissi, Junie Flore Yimga, Désiré Takou, Sylvie Moudourou, Marinette Ngo Nemb, Jean-Bosco Nfetam Elat, Maria-Mercedes Santoro, Carlo-Federico Perno, Vittorio Colizzi, Alexis Ndjolo

**Affiliations:** 1 Chantal BIYA International Reference Centre for Research On HIV/AIDS Prevention and Management (CIRCB), Melen Road, PO BOX 3077, Yaounde, Cameroon; 2 Faculty of Medicine and Biomedical Sciences, University of Yaoundé I, Yaoundé, Cameroon; 3 National HIV Drug Resistance Working Group, Ministry of Public Health, Yaounde, Republic of Cameroon; 4 Department of Experimental Medicine, Faculty of Medicine and Surgery, University of Rome Tor Vergata, Rome, Italy; 5 Central Technical Group, National AIDS Control Committee, Yaoundé, Cameroon; 6 Department of Microbiology, University of Milan, Milan, Italy; 7 UNESCO Multidisciplinary Board of Biotechnology, University of Rome Tor Vergata, Rome, Italy; 8 Faculty of Biomedical Sciences, Evangelic University of Cameroon, Bandjoun, Cameroon

**Keywords:** HIV/AIDS, Virological success, ART duration, Test and treat era, Cameroon

## Abstract

**Background:**

After the launching of the « *Test & Treat *» strategy and the wider accessibility to viral load (VL), evaluating virological success (VS) would help in meeting the UNAIDS targets by 2020 in Cameroon.

**Setting and methods:**

Cross-sectional study conducted in the Chantal BIYA International Reference Centre for research on HIV/AIDS prevention and management (CIRCB), Yaoundé, Cameroon; data generated between October 2016 and August 2017 amongst adults, adolescents and children at 12, 24, 36 and ≥ 48 months on ART. VS was defined as < 1000 copies/mL of blood plasma and controlled viremia as VL < 50 copies/mL. Data were analysed by SPSS; p < 0.05 considered as significant.

**Results:**

1946 patients (70% female) were enrolled (1800 adults, 105 adolescents, 41 children); 1841 were on NNRTI-based and 105 on PI-based therapy; with 346 patients at M12, 270 at M24, 205 at M36 and 1125 at ≥ M48. The median (IQR) duration on was 48 months (24–48). Overall, VS was 79.4% (95% CI 77.6–81.2) and 67.1% (95% CI 64.9–69.1) had controlled viral replication. On NNRTI-based, VS was 79.9% vs. 71.4% on PIs-based, p = 0.003. By ART duration, VS was 84.1% (M12), 85.9% (M24), 75.1% (M36) and 77.2% (≥ M48), p = 0.001. By age, VS was 75.6% (children), 53.3% (adolescents) and 81.1% (adults), p < 0.001.

**Conclusions:**

In this sub-population of patients receiving ART in Cameroon, about 80% might be experiencing VS, with declining performance at adolescence, with NNRTI-based regimens, and as from 36 months on ART. Thus, improving VS may require an adapted adherence support mechanism, especially for adolescents with long-term treatment in resource-limited settings.

## Introduction

Despite many decades of continuous fight, Human immunodeficiency virus (HIV) is still one of the major global health issue, having claimed more than 35 million lives so far, with the WHO African Region in particular being the most affected with 25.7 million people living with HIV in 2017 [1, 2]. As the momentum in the efforts to control the pandemic rises, the global commitment to ending HIV/AIDS epidemic was set by the United Nations (UN) Assembly for 2030 [3]. Reducing the incidence and providing antiretroviral treatment to the infected people are key in the progress and achievement of this goal. A great stride in the journey towards ending HIV/AIDS is the ambitious treatment targets set by the Joint United Nations Programme on HIV/AIDS (UNAIDS), the 90–90–90 strategy by 2020. This goal stipulates that by 2020, 90% of all people living with HIV will know their HIV status; 90% of all people with diagnosed HIV infection will receive sustained antiretroviral therapy; and 90% of all people receiving antiretroviral therapy will have viral suppression [1, 4].

Achieving these targets is especially challenging for developing countries where limited access to health care, drug availability and adequate provision of viral load monitoring tools and other programmatic issues need to be addressed. The 2017 report of the UNAIDS on ending AIDS progress reveals that globally, 70% of infected people know their HIV status, 77% of these are receiving combination antiretroviral therapy (cART) and 82% of treated patients have virological success (VS) [4]. Studies conducted in some developing countries, such as Cameroon reported a viral suppression level less than 80% [5–8]. Apart from the recent Cameroon Population-based HIV impact assessment (CAMPHIA) [9], most of these studies [6, 7] were conducted before the implementation of the “*test and treat”* strategy. In addition, since most of the studies focused on adult populations [5, 9], data on viral suppression among children and adolescents in Cameroon are scare.

Thus, we decided to investigate on the viral suppression levels according to different age groups, therapeutic regimen and duration on ART in the “*test and treat”* era which is characterized by a wider accessibility to viral load testing in Cameroon.

## Methods

### Study design and setting

This is a retrospective cross-sectional study conducted from October 2016 to August 2017, which corresponds to the effective start of “*test and treat*” strategy in Cameroon. The study population was made up of children, adolescents and adults who are routinely monitored for viral load testing at the Chantal BIYA International Reference Centre for research on HIV/AIDS prevention and management (CIRCB). The CIRCB routinely receives and tests viral load (VL) samples mostly from 3 regions (the Centre, the South and the East regions of Cameroon; according to the allocation of HIV follow up units, to laboratories included in the viral load testing network by the Ministry of Public Health) out of the 10 existing regions of the country. The patients considered for this study were received from a total of 54 health facilities. However, majority of the samples were from Yaoundé, the city capital. Referring health facilities are district hospitals (349 patients), regional hospitals/central hospitals (660 patients), general hospitals (601 patients), private hospitals (271 patients) and other types of health centres (65 patients). Treatment history was collected from their medical records.

### Viral load quantification

HIV-1 RNA quantification was performed on plasma samples using the *Abbott m2000rt RealTime HIV*-platform according to manufacturer recommendations (Abbott Molecular Inc. 1300 E. Touhy Ave. Des Plaines, IL 60018 200680-105; USA). A protocol using 0.6 mL of plasma was used for RNA extraction. The lower limit of detection of the assay is < 40 copies/mL of HIV-1 RNA. This laboratory is registered with two viral load proficiency-testing programs. The study received institutional approvals from both National AIDS Control Committee (NACC) and the Chantal BIYA International Reference Centre for research on HIV/AIDS prevention and management (CIRCB).

### Inclusion criteria

All patients with complete information on date of sample collection, age, date of ART start and current ART regimen; together with a VL result were included. Patients on treatment for less than 12 months were excluded from our analysis. Data were queried from the data base and was cleaned. Age groups were defined as follows: children (0–9 years), adolescents (10–19 years) and adults ≥ 20 years. Virological success (VS) was defined as viral load (VL) < 1000 RNA-copies/mL of blood plasma, virological failure (VF) as VL ≥ 1000 RNA-copies/mL [10] and very low level viremia < 50 copies/mL [11] was considered as controlled viremia.

### Data analysis

All data were analysed using SPSS *version 20.0* (SPSS Inc., Chicago, Illinois), with a statistical significance level set at p < 0.05. Frequencies, proportions, confidence interval were computed and data were summarised using tables and figures. Hypothesis testing was performed using Pearson Chi Square and Chi Square for trends as appropriate.

## Results

### Participants’ characteristics

Table 1 shows the characteristics of our study population. A total of 1946 patients were enrolled, all reported to be naïve to cART at the moment of treatment initiation. The majority was female (1373; 71%) were enrolled in this study. The median (interquartile range: IQR) age of our study sample was 41 years (IQR: 34–50 years); the median year of cART start was 2012 (IQR: 2009–2014); and the median duration on treatment was 48 months (IQR: 24–48 months). Most patients were adults (92.5%) and 89.3% lived in an urban area. Among the 1841 patients on first line antiretroviral therapy, most patients 1017 were on tenofovir + lamivudine + efavirenz (TDF + 3TC + EFV) combination. Out of the 1946 patients, 49.7% was diagnosed following a consultation, against 28.2% in voluntary screening and 15.3% of females during PMTCT (protection of HIV transmission from mother to child program).

**Table 1:** Population characteristics and viral suppression levels

Variable	Overall^a^ N = 1946	< 50 copies/mL^b^ N = 1305 (67.1%)	< 1000 copies/mL^b^ N = 1546 (79.4%)	≥ 1000 copies/mL^b^ N = 400 (20.6%)	p-value*
Age in year, median (IQR)	41 (34–50)	41 (34–50)	42 (34–50)	39 (33–49)	< 0.001
Year of cART start, median (IQR)	2012 (2009–2014)	2012 (2009–2014)	2012 (2009–2014)	2011 (2008–2013)	< 0.001
Duration on cART in month, median (IQR)	48 (24–48)	48 (24–48)	48 (24–48)	48 (36–48)	0.001
Gender, n (%)					
Male	573 (29.4)	352 (61.4)	435 (75.9)	138 (24.1)	0.013
Female	1373 (70.6)	953 (69.4)	1111 (80.9)	262 (19.1)	
Age groups, n (%)					
Children	41 (2.1)	25 (61.0)	31 (75.6)	10 (24.4)	
Adolescents	105 (5.4)	46 (43.8)	56 (53.3)	49 (46.7)	< 0.001
Adults	1800 (92.5)	1234 (68.6)	1459 (81.1)	341 (18.9)	
Therapeutic regimen, n (%)					
First line	1841 (94.6)	1261 (68.5)	1471 (79.9)	370 (20.1)	0.037
Second line	105 (5.4)	44 (41.9)	75 (71.4)	30 (28.6)	
ARV combination, n (%)					
*TDF *+ *3TC *+ *EFV*	1070 (55.0)	770 (72.0)	890 (83.2)	180 (16.8)	< 0.001
Other 1st line ARV^c^	771 (39.6)	491 (63.7)	581 (75.4)	190 (24.6)	
ATVr or LPVr Based ARV^d^	105 (5.4)	44 (41.9)	75 (71.4)	30 (28.6)	
Circumstance of diagnosis, n (%)					
Consultation	967 (49.7)	625 (64.6)	750 (77.6)	217 (22.4)	< 0.001
Voluntary	549 (28.2)	396 (72.1)	461 (84.0)	88 (16.0)	
PMTCT	210 (10.8)	153 (72.9)	180 (85.7)	30 (14.3)	
At birth	41 (2.1)	22 (53.7)	25 (61.0)	16 (39.0)	
Other/unknown	179 (9.2)	109 (60.9)	130 (72.6)	49 (27.4)	
Urban vs rural, n (%)					
Urban	1737 (89.3)	1171 (67.4)	1388 (79.9)	349 (20.1)	0.004
Rural	144 (7.4)	98 (68.1)	117 (81.2)	27 (18.8)	
Unknown	65 (3.3)	36 (55.4)	41 (63.1)	24 (36.9)	

*cART* combined antiretroviral therapy, *ARV* antiretroviral, *TDF* tenofovir, *3TC* lamivudine, *EFV* efavirenz, *ATVr* ritonavir boosted atazanavir, *LPVr* ritonavir boosted lopinavir, *PMTCT* prevention from mother to child transmission, *IQR* interquartile range

^a^Percentages in this column represent column percentage

^b^Percentages in this column represents row percentage

^c^Other first line ARV [3TC + d4T + NVP (n = 1), ABC + 3TC + EFV (n = 12), ABC + 3TC + NVP (n = 5), AZT + 3TC + EFV (n = 51), AZT + 3TC + EFV (n = 486), TDF + 3TC + NVP (n = 216)

^d^Lopinavir based (n = 31) and atazanavir based (n = 68). * p-value for virological success at < 1000 copies/mL

### Prevalence of viral suppression

The overall prevalence of VS after at least 12 months on cART at VS < 1000 copies/mL and VS < 50 copies/mL was 79.4% (95% Confidence interval, CI 77.6–81.2) and 67.1% (95% CI 64.9–69.1) respectively. The median age, median year of cART initiation, and median duration on cART for patients failing treatment vs. those on VS at ≥ 12 months of cART were: 39 [IQR: 33–49] years vs. 41 [IQR: 34–50] years, p < 0.001; calendar year 2011 [IQR: 2008–2013] vs. calendar year 2012 [IQR: 2009–2014], p < 0.001; and 48 [IQR: 36–48] months vs. 48 [IQR: 24–48] months, p = 0.001; respectively.

According to ART duration, VS was 84.1% at 12 months (M12), 85.9% at 24 months (M24), 75.1% at 36 months (M36) and 77.2% at more 48 months (≥ M48), p = 0.001. The overall VS was 75.9% (95% CI 72.3–79.2) for males and 80.9% (95% CI 78.8–82.9) for females, p = 0.013; while overall controlled viremia was 61.4% for male and 69.4% for female (p = 0.001). There was a large variation of VS prevalence with respect to age groups for both VS thresholds (p < 0.001); with the highest prevalence of virological failure at VS ≥ 1000 copies/mL being recorded among adolescents (46.7%), followed by children (24.4%). When compared according to cART regimens, TDF + 3TC + EFV, others first line combinations, and ritonavir-boosted lopinavir (LPV/r)/atazanavir (ATV/r)-based ARV) at VS < 1000 copies/mL, patients on TDF + 3TC + EFV recorded the highest VS (83.2%) versus 71.4% on PI/r-based regimens, p < 0.001.

**Fig. 1:**
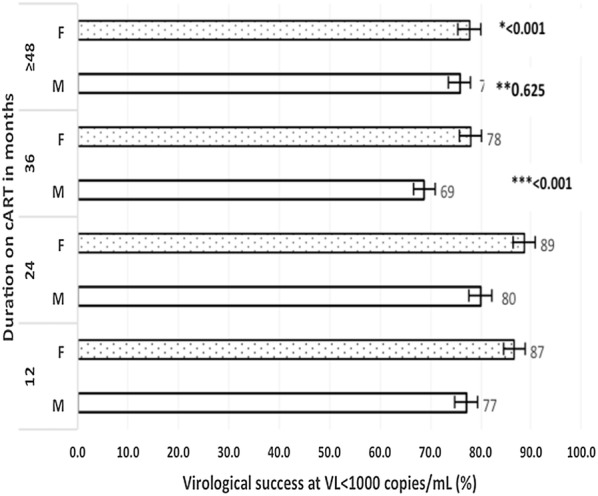
On-treatment virological success per duration on cART and per gender. *cART* combination antiretroviral therapy, *VL* viral load, *F* female, *M* male. *p-value for trend of virological success per duration on cART and per female gender; **p-value for trend of virological success per duration on cART and per male gender; ***overall p-value for trend of total population. Error bars represent 95% confidence interval

**Fig. 2:**
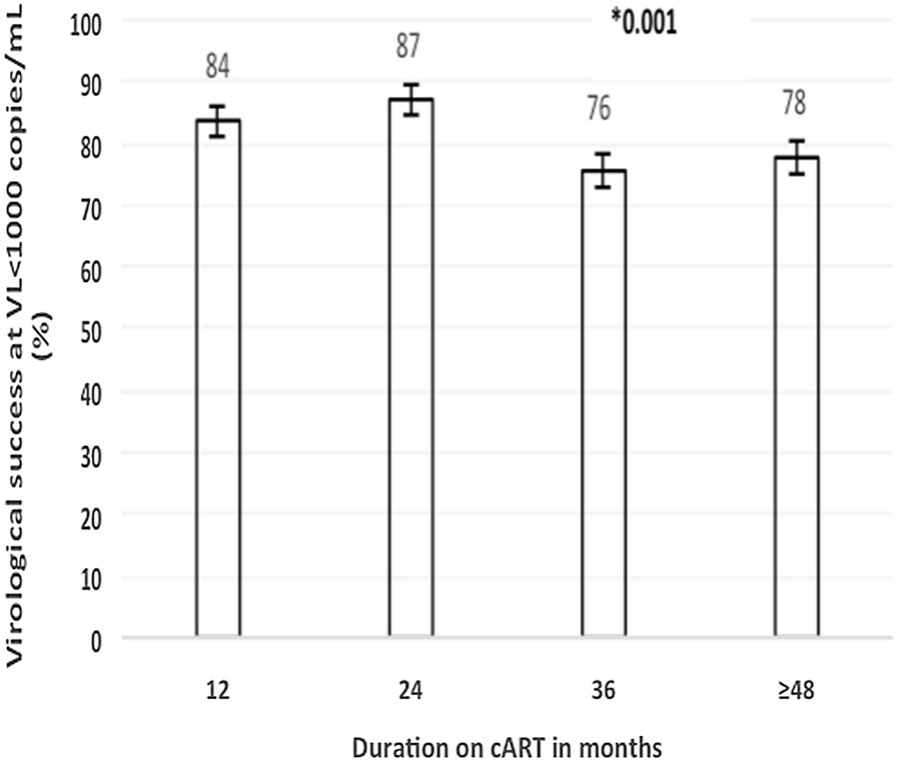
On-treatment virological success per duration on 1st line-NNRTI-based cART. *cART* combination antiretroviral therapy, *VL* viral load, *1st line* first line therapy-NNRTI-based. *p-value for trend of the 1st line over time. Error bars represent the 95% confidence interval

According to circumstances of HIV diagnosis, at both VS < 50 copies/mL and VS < 1000 copies/mL, those diagnosed during PMTCT had the highest prevalence (72.9% and 85.7% respectively), followed by patients screened voluntarily (72.1% and 84.0% respectively); with patients diagnosed at birth recording the worst performance (53.7% and 61.0% respectively); p < 0.001. Figure 1 shows that for VS < 1000 copies/mL per duration on cART and per gender, the prevalence ranged from 69% to 80% (at 36 and 24 months respectively) for male (p = 0.625); against 78% to 89% (≥ 36 months and 24 months respectively) for female (p < 0.001). On the other hand, Fig. 2 shows that for the same VS level per duration on cART and per 1st line NNRTI (non-nucleoside reverse transcriptase inhibitor)-based therapy, it ranged from 76% to 87% (at M36 and M24 respectively), p = 0.001).

## Discussion

In this study, we aimed at estimating the prevalence of virological success per age groups, duration on cART and therapeutic line among HIV/AIDS patients in Cameroon. It shows that at the start of the “*test and treat*” era in Cameroon, the overall prevalence of VS < 1000 copies/mL after at least 12 months of cART was 79.4% (95% CI 77.6–81.2); about 11% away from the 90% target set by UNAIDS by 2020. This performance is less than the 82% reported global VS performance [4]. In the other hand, the prevalence of patients with controlled viremia after at least 12 months of cART was 67.1% (95% CI 64.9–69.1), far away from the high VS reported in many western countries [12]. The virological failure rate of 20.6% observed in this study was within the range reported in other developing countries, which is 3.7% to 26.0% [7]. Previous studies in Cameroon reported VS between 72.1 and 90.2% [5, 6, 9, 13], with the differences in VS performance mostly attributed to differences in study population characteristics and duration on cART. Our result is closely similar to the recent and country wide population based HIV impact assessment (CAMPHIA) study which found 80% VS. The high VF in this study may be either related to the fact that many patients might be treatment failure suspects and/or repeat testers after suspect failure, which are known to record a high VF compared to patients on routine monitoring [14]; or to a relatively higher proportion of children and adolescents (compared to other studies in Cameroon), who generally have a low response [9, 14–19], especially in settings with a weak health system.

The median age (IQR) of virally suppressed patients was 42 years (34–50 years) versus 39 years (33–49 years) in treatment failure patients (p < 0.001). In fact, our result shows that children (< 10 years) and/or adolescents (10–19 years) are much less likely to achieve virological success compared to adult populations (p < 0.001). Poor ART response of HIV-infected children (especially infants perinatally infected) compared to adult populations is well documented. This could be justified by the higher viral replication and the less efficient immune response against infections in infants [20–22]. Moreover, patients with pre-therapy viral load of > 500,000 copies/mL (commonly observed in children) are known to have longer time to VS and a higher probability of virological rebound after VS [23]. Nonetheless, suboptimal adherence level is also a major challenge often reported in children [24].

The lack of adherence and adequate provision of psychological supports in children and adolescents has been reported to represent the major cause of loss to follow-up and virological failure [14, 24]. The VS performance observed in our study in adolescent population in particular is a call for concern. It was also recognized in some settings that they have a limited access to antiretrovirals (ARVs) [3]. Thus, there is a need to give a special considerations to these vulnerable groups in the provision of health care delivery. In addition, they should have more access to drug resistance testing because they have been reported to record generally a high burden of HIV drug resistance [25, 26].

According to gender, females are more likely to experience virological success than men 80.9% vs 75.9% respectively, p = 0.013). Even though the recent CAMPHIA study reported a relatively higher VS in men than women (80.1% vs 79.2%) [9], data from many studies suggest that men are likely to experience virological failure than females [4, 15]. This can be justified by their high risk related behavioural patterns. For example, studies reported that the masculine gender norms contribute to greater risk-taking; expressing manhood by having multiple sex partners, refusal to use condoms, alcohol and substances abuse; and poorer uptake of health services [27–30]; all leading to a poorer adherence and treatment interruption which favour treatment failure. In addition, it is recognised especially in some Sub-Saharan Africa that men are most likely to die of HIV/AIDS than women [27, 30] because they have lower knowledge of HIV/AIDS [16], and present generally to health service latter with advanced disease conditions [30–32]. While sustaining and improving the access to ARV and VS in women through existing programs, men should not be left behind. Since current health system design may be responsible for these gaps, designing interventions separately for males and females, filling the gaps in the continuum of HIV/AIDS care [33] and increasing case finding through PMTCT, index and work place testing [4, 28, 30] may help achieve the 90% target UNAIDS goal.

The treatment failure rate at VS < 1000 copies/mL was higher among patients on second line compared to first line patients (28.6% vs 20.1% respectively, p = 0.037). When compared according to ARV combinations (TDF + 3TC + EFV, others first line combinations or protease inhibitors-based ARV, patients on TDF + 3TC + EFV recorded the highest virological success (83.2%) versus 75.4% and 71.4% for other first line and PI-based regimen respectively (p < 0.001). Most studies reported more than 80% VS in patients on PI based therapy [34–36]. Even though low rate of switching from first to second line, which may affect second line response is sometimes reported [37], Protease inhibitors (PIs) based regimens are generally protective against VF [19]. The small number of patients (105) on second line in our study may not be representative; however, this may indicate adherence challenges among patients in second line treatment. Our result also suggests that there is a better response to TDF + 3TC + EFV combination compared to other first line options (including those that are nevirapine (NVP)-based and those containing zidovudine (AZT), stavudine (d4T) and abacavir (ABC) as NRTI-backbone). In fact, this combination has been found to be equivalent or superior to its comparator arms (other nucleoside reverse transcriptase (NRTI)—backbone and/or NNRTI) in many studies [38–41].

According to duration on therapy, VF was associated with longer stay on cART (median year of cART start: 2011 (2008–2013) versus 2012 (2009–2014 for VF and VS group respectively, p < 0.001). Similar trend was equally reported after 6 months and 48 months on cART in a review [8]. The individual increase in the lack of tolerability and the emergence of multi strain viruses with time greatly account for this reduced VS [6, 14, 42, 43]. A study in Cameroon reported that the prevalence of VF and resistance increased with time on ART, from 12.0 to 8.0% in the 6- to 12-month group to 31.3% and 27.1% in the > 72-month group, respectively [5]. Unavailability of ART at the treatment centre was reported as the single most common cause for incomplete adherence in rural Cameroon [44]; strategies to improve adherence through health system strengthening should be implemented. To limit the emergence of viral resistance and achieved a higher and sustained VS, the use of novel drug classes such integrase strand transfer inhibitors (INSTI) class in first line, which has a demonstrated excellent efficacy and resistance profile in clinical practice, and recommended by many guidelines today will be a good option [43, 45–48]. Figure 1 suggests that the variation of VS over time in females showed a significant level (p < 0.001), while variation in males was not significant (p = 0.625). Gender differences in HIV disease progression and treatment outcomes among females and males [49] as well as other socio cultural characteristics, behavioural differences and disease perception may account for such differences.

According to reasons/circumstances of diagnosis, the VS varied significantly among the patients (p < 0.001). The VF among patients diagnosed during consultation was 22.4%, against 16.0% in patients who undertook voluntarily testing and 14.3% in women diagnosed during PMTCT. Additional data should be collected to better understand how engaging potentially infected persons and key populations in voluntary screening may contribute in achieving also the third “90”. Contrary to other studies, we noted a higher VS among patients from Rural area compared to those in urban area (p = 0.004). The major limitation of this study is that data were not collected on whether VL was requested for routine monitoring, suspects failure or repeat testers after failure, and might have confounded our estimates. Additionally, even though the acquired HIV drug resistance among patients failing ART is still of concern in Cameroon, we could not present these data in our study because they were not performed. We therefore recommend subsequent studies to investigate other predictors to VF and acquired HIV drug resistance profile among patients failing ART in such setting.

## Conclusion

In this sub-population of patients receiving ART in Cameroon, about 8 out of 10 might be experiencing VS (with a gap of 10% below the required target set by 2020), with poorer outcomes among adolescents and those as from 36 months on cART. Furthermore, VS appears higher in females and in those treated with TDF + 3TC + EFV compared to other NNRTI-based or PI/r-based regimens. Thus, strategies towards improved monitoring of adolescents, male and long-term treated patients are crucial in maximising VS, while access to timely switch of ART and/or drug resistance would help in alleviating the burden VF and in meeting the target for ART response in RLS.

## Data Availability

Data supporting the findings are fully available in the results, in the tables and figures of the manuscript.
